# The Effect of Psycho-Education Intervention Based on Relaxation Methods and Guided Imagery on Nausea and Vomiting of Pregnant Women 

**Published:** 2019-03

**Authors:** Mansour Shakiba, Homeyra Parsi, Zahra Pahlavani Shikhi, Ali Navidian

**Affiliations:** 1 Department of Psychiatry, School of Medicine, Zahedan University of Medical Sciences, Zahedan, Iran; 2 Department of Midwifery, School of Nursing and Midwifery, Zahedan University of Medical Sciences, Zahedan, Iran; 3 Community Nursing Research Center, Zahedan University of Medical Sciences, Zahedan, Iran

**Keywords:** Pregnancy, Nausea, Vomiting, Relaxation, Psycho-Education, Women

## Abstract

**Objective:** The present study aims at evaluating the effects of psycho-education based on relaxation methods on Hyperemesis Gravidarum (HG).

**Materials and methods:** This is a quasi-experimental study, with pretest and posttest design, which was carried out on 100 pregnant women with complaints of nausea and vomiting who had referred to general health centers to receive pregnancy care. In accordance with the specified content, women in the intervention group received 3 sessions of psycho-education based on relaxation methods for a week. Four weeks after the end of education and before the 16^th^ week of pregnancy, data were gathered from both the intervention and control groups using the Pregnancy-Unique Quantification of Vomiting and Nausea (PUQEN) scale. Data were analyzed with the aid of statistical tests.

**Results:** There was no significant difference between the average values of HG in the intervention group and the control group prior to the intervention. However, after receiving psycho-education, the average value of nausea and vomiting in pregnant women of the intervention group (5.11 ± 1.60) was significantly lower than that in the control group (6.00 ± 1.66) (p = 0.035).

**Conclusion:** The psycho-education based on relaxation methods of this study had a positive and significant effect on reducing the intensity of HG. It is helpful to integrate the educational content of this intervention in the caring programs of pregnant women with nausea and vomiting.

## Introduction

Pregnant women, undergoing one of the most sensitive periods of their life, experience symptoms such as nausea and vomiting (1). Pregnancy nausea and vomiting, technically known as Hyperemesis Gravidarum (HG), can start a short while after the beginning of pregnancy, such that nausea has come to be known as one of its indicative signs (2). Symptoms usually emerge between the 4^th^ and the 6^th^ week of gestation, reach their peak between the 8^th^ and the 12^th^ week, and recede between the 16^th^ and the 20^th^ week (3). About 20 to 30%of women may experience these symptoms throughout the entire pregnancy duration (4). In most cases, factors such as mobility, a burning sensation in the stomach, consumption of certain types of food, or certain aromas trigger the symptoms. HG is not always caused by pregnancy as such since other possible factors are also involved (5). The prevalence of HG varies between 35 to 91%in different parts of the world, and severe forms of this complication are less common. Thus, it is estimated between 60.6 to 72.9% in Turkey and an average of 69.7%in Iran (6, 7). 

The unpleasant feeling of nausea and vomiting can be the source of considerable distress, discomfort, and temporary disability even when the symptoms are not very severe. Women’s everyday activities begin to change, and most of their time and energy is spent concentrating on nausea and vomiting. They feel more tired and in need of more rest. HG also has negative psychological effects on women. They suffer from physical, social, and emotional isolation due to a sense of guilt and the fear of losing control and needing help. Women lose their confidence and are constantly afraid of getting sick. Out of the fear of vomiting in front of others, they have difficulty performing their routine tasks at home or at work (3, 2, 8). HG cause impatience, irritability, anxiety, sleep disorders, and psychological turbulence, and it has adverse effects on the familial, social and professional aspects of life (9). There is also a relation between HG and intrauterine fetal growth restriction (10). On the other hand, babies born of mothers with this condition are more agitated and colic, and in comparison to other infants, they are more likely to develop sleep, alimentation, and excretion problems. These infants are faced with more learning problems, delay in puberty, irritability, and distraction in their childhood (11). 

The causes of HG are still unknown and it is commonly defined as a multi-factorial condition. Various biological, psychological, and socio-economic factors are involved in its pathology. HG has a psychological aspect, since it is very common in women with immature personality, unintended pregnancy, pregnancy anxiety and depression, histrionic personality disorder or hysteria, conversion hysteria disorder, neurotic disorders, and psychosis. Since the relationship between nausea and vomiting and stress in pregnant women has been identified, the hypothesis that psychological factors play a significant role in HG is reaffirmed (12, 4). Women’s psychological status is a very influential factor in causing pregnancy nausea and vomiting. Fear, rejecting pregnancy and refusing to take on the mother’s role, unintended pregnancy, and anxiety manifest themselves by nausea and vomiting. Nausea and vomiting are controlled by the nervous system, and anxiety may play a role in activating the condition (13).

At present, there are some medical approaches to eliminate or reduce HG symptoms, but there is no empirical evidence to prove their efficiency. On the one hand, women have concerns about any sort of pharmacological intervention, since they do not know the full effect of such medication on their health and that of their fetus. That is why they prefer to control the condition by personal, familial, and traditional coping strategies, as well as focusing on non-pharmacological methods that professionals may prescribe (8). Women seek coping strategies for this problem on their own. Therefore, helping them to deal more effectively with this condition and the negative anxieties it entails during pregnancy is one of the most central objectives of pregnancy care (14). Davis (2004) believes that one must begin by non-pharmacological methods and eventually move towards medication (15). 

Since the etiology of HG is not yet fully known, therapeutic approaches do not correspond toits etiology; instead, they aim at reducing or eliminating the symptoms (2). By the same token, finding an effective and definitive treatment is complicated (1). Concerns relating to the hazardous effects of medications on the fetus have led to many pregnant women’s rejection of receiving any sort of medical treatment for nausea and vomiting. They are willing to either tolerate the discomfort caused by the condition, or look for a safe, non-pharmacological treatment. Some women even express worries concerning the alternative treatments of herbal products (3, 14). Different methods of complementary and alternative medicine are highly popular in the society especially amongst pregnant women, because when it comes to their health and that of their fetus, pregnant women primarily hold themselves responsible. On the other hand, they believe such methods to be natural and, therefore, harmless (8). 

Various non-pharmacological methods including psychotherapy coupled with medical treatments (Faramarzi et al. 2015), acupressure (Gurkanand Arsalan, 2008), and counseling (Isbirand Mete, 2016) have been used to reduce the severity of HG symptoms (16, 17, 8). One of the complementary, non-pharmacological treatments is relaxation. Even though progressive muscle relaxation (PMR) and guided imagery are considered complementary medicine and alternative treatment, they are categorized as cognitive behavioral strategies (18). Strategies such as stress reduction and guided imagery have already been used to control nausea and vomiting in patients taking chemotherapy (19-21). PMR is a care intervention defined as a procedure to facilitate consecutive contraction and relaxation in a group of muscles, which simultaneously leads to the recognition of different sensations. This method has been proven effective in coping with nausea and vomiting (22). Due to the relation that exists between HG severity and women’s perceived stress level (23), integrating stress-reduction methods of PMR and guided imagery in common pregnancy training programs could be effective in controlling nausea and vomiting. 

In most cases, women who suffer from pregnancy nausea and vomiting do not receive proper care and the necessary medical attention. Nausea and vomiting might be a common complication during pregnancy and might gradually disappear on its own. However, because this condition causes numerous problems for women and their families, researchers have carried out extensive studies in search of some solutions. In conventional coping strategies and therapeutic methods used to mitigate HG, psychological and social aspects of women’s lives are not adequately taken into consideration. Psychological factors involved in this condition such as anxiety, emotions, psychological adaptation, acceptance of pregnancy, and the psychological significance of symptoms are generally ignored as well. Consequently, it is necessary to design and examine interventions which integrate relaxation methods and conventional trainings in order to deal with pregnancy symptoms while taking into account the psychological distress of pregnant women. Due to the high rate of acceptability and popularity of non-pharmacological treatments amongst pregnant women, the present study aims at determining the efficiency of psycho-education based on relaxation methods on pregnant women’s nausea and vomiting.

## Materials and methods

This is a quasi-experimental study involving a pretest and posttest design and a control group. All pregnant women with HG complaints who referred to general health centers in 2018 in order to receive pregnancy care were consisted the study population. The size of population under study was determined with an accuracy of 95 % and statistical test power of 90%, in accordance with mean and standard deviation of the values of nausea and vomiting in the study of Isbirand Mete (2016). Taking into account the possibility of people leaving the study, the size of each group was estimated at 50, which makes a total of 100 for the two study groups. The participants were chosen out of pregnant women with HG by means of convenience sampling; then, they were randomly divided into two groups. The inclusion criteria were: moderate severity of nausea, history of HG in at least three days, HG occurring between the 4^th^ and the 6^th^ week of pregnancy, no multiple pregnancy or any other condition that would increase HCG, no underlying medical condition in the current pregnancy, no use of antiemetic, no historyof traumatic or distressing event during the current pregnancy, and 18+ years of age. The exclusion criteria, on the other hand, were: abortion, no increase in the severity of nausea and vomiting and hyperemesis, taking drugs during the study including prescription by a physician and self-medication, and being absent from more than one training session. 

Data collection in this research was performed using a two-part questionnaire which was used after its reliability was established. The first part included questions on personal information and data concerning the current pregnancy, and the second part included questions about nausea and vomiting. The Pregnancy-Unique Quantification of Vomiting and Nausea (PUQE) scale was designed and developed by Koren et al., and it includes three questions related to the condition of nausea, vomiting, and retching each question designed with 5 options on the Likert scale. Thus, the scaling range of this device falls between a minimum of 3 and a maximum of 15. Based on the scores achieved by the pregnant women who completed this questionnaire, the participants were divided into four groups of ‘no symptom’ (a score under 4), ‘mild symptoms’ (a score of 4 to 6), ‘medium symptoms’ (a score of 7 to 12), and ‘severe symptoms’ (a score of 13 or above) of nausea and vomiting. The validity and reliability of this device have been evaluated in many studies (24, 25). In Iran, these characteristics have been examined and confirmed by Soltani et al., and the scale has been subsequently employed in other studies such as Nikibakhsh et al. (2016) (9). 

After receiving an official letter of introduction and visiting the general health centers, the authors made the necessary arrangements with the related authorities in order to ask them to cooperation carrying out the research. 

**Figure 1:**
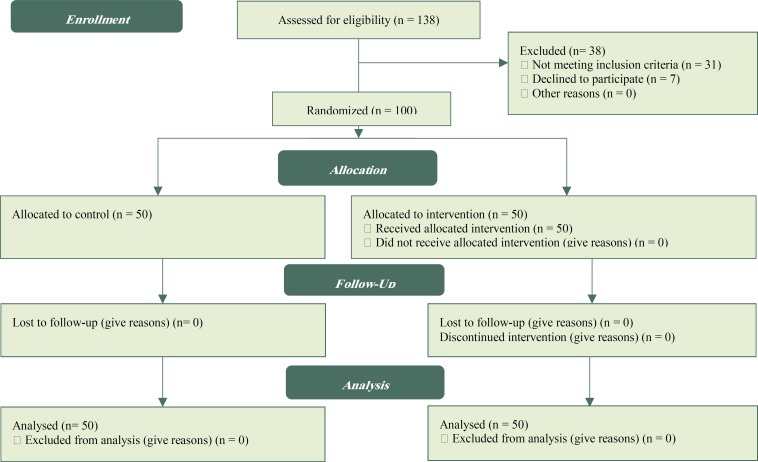
Flow chart of participants through each stage of randomized clinical trial

Initially, with the help of PUQE questionnaire and interviews, pregnant women with a medium severity of nausea and vomiting were identified. These were women with scores between 7 and 12 who met the necessary qualifications to enter this research. A total of 138 eligible women were examined, 38 individuals (31 due to not meeting inclusion criteria, 7 due to decline to participate) were excluded. As a result, the study was conducted and followed up on 100 women (Figure 1). A written consent form was taken from all qualified participants. The selected mothers were then randomly divided into two groups of intervention and control. The process of random selection is described as follows: 100 balls were prepared, corresponding to the total number of participants. The balls were in two colors: red assigned to the intervention group, and white assigned to the control group. All balls were placed inside a vase; the participants were enlisted one by one in their respective group based on the order of the ball randomly taken out of the vase. A list was prepared which determined the group to which each participant belonged. In the pretest, all participants in both groups completed the questionnaire which determined the severity of nausea and vomiting. For one week, participants in the intervention group individually received 3 sessions (each lasting between 60 to 90 minutes) of psycho-education based on relaxation methods and in accordance with the specified content (Table 1). 

**Table 1:** The structure of the sessions and the content of the psycho-educational program

**Session**	**The content of the session**
First	familiarization, introduction and statement of the group rules, review of the physiological and psychological changes in pregnancies that generate nausea and vomiting + instructing diaphragmatic breathing and progressive muscle relaxation exercises (practical teaching and use of instructional CD)	
Second	Modification of underlying stimuli: identification of nausea and vomiting triggers, adjusting lifestyle and nutritional instructions + breathing exercises, demonstrating and practicing guided imagery (guided imagery instructional CD)	
Third	Acceptance of emotions: self-expression, recognition of emotions and thoughts, reinforcing positive emotion + muscle relaxation and guided imagery training.	

In order to ensure that participants applied the instruction scorrectly, each woman was also given a checklist of the relaxation exercises to complete.

After 4 weeks as the posttest, participants were asked to re-fill the questionnaires at home or in the health center. During the 4 weeks of study, the researchers made an effort to keep in touch with all participants by calling them to answer any possible questions they might have. Women in the control group received no additional education except for the usual pregnancy care, and they completed the same questionnaires 4 weeks post-intervention in order to take the posttest. Of course, after the research was over, women of the control group received the compact disc of the training program.

After reviewing the literature on relevant interventional approaches and clinical trials, the authors designed the first format of psycho-education with an emphasis on the suggestions of Isbirand Mete (2016), Isbirand Mete (2010), Nakamura (2010), and Baghdari et al. (2016) (6, 8, 26, 27). Then, to increase the scientific validity of this study, consultations were made with relevant health professionals including counselor or clinical psychologists, obstetricians, and midwifery specialists. Based on the opinions of these experts, the initial model was adjusted and the final protocol was prepared. The intervention was implemented by a Master of Midwifery Counseling with clinical experience under the supervision of a specialist with a Ph.D. in Counseling Psychology. 

Paired T-test was used to compare pre and post-intervention means. Independent t-test was later used to compare mean score between intervention and control groups. The significance level in this study was considered to be 0.05.

This study was approved by the Ethics Committee of Zahedan University of Medical Siences with the Ethic code of IR.ZAUMS.REC.1379.205. Providing information on the process and time of research, the type of intervention and the need for written informed consent, were among ethical considerations observed in this study.

## Results

Concerning the demographic characteristics of pregnant women, as shown in table 2, the average age of women in the intervention group and the control group was respectively 25.10 ± 5.83 and 25.50 ± 6.27 years. 95% of participants in the intervention group and 97.5% of participants in the control group were unemployed women or housewives. Only 15% of the intervention group and 10% of the control group had academic education.

**Table 2:** Demographic characteristics of the intervention and control groups

	**Intervention** **n (%)**	**Control** **n (%)**	**P-value**
Women's occupation			
Housewife	46 (92)	46 (92)	0.99
Employed	4 (8)	4 (8)	
Total	50 (100)	50 (100)	
Women's education			
Illiterate	9 (18)	14 (28)	0.005
Lower than diploma	33 (66)	17 (34)	
Higher than diploma	8(16)	19(38)	
Total	50 (100)	50 (100)	
Pregnancy			
Wanted	23 (46)	21 (42)	0.68
Unwanted	27 (54)	29 (58)	
Total	50 (100)	50 (100)	
History of domestic violence			
Yes	40 (80)	37 (74)	0.47
No	10 (20)	13 (26)	
Total	50 (100)	50 (100)	
	**Mean ± SD**	**Mean ± SD**	
Age of women (Year)	28.58 ± 4.47	29.08 ± 5.03	0.52
Gestational age (Week)	22.88 ± 1.68	22.16 ± 1.60	0.03
Marriage duration (year)	9.00 ± 5.12	9.06 ± 4.68	0.06

**Table 3:** Nausea and vomiting Scores in Intervention and Control pregnant women before and after the psycho-education

	**Before** **Mean ± SD**	**After** **Mean ± SD**	**Changes** **Mean ± SD**	**Paired t ** **test(Before-After)**
Control	8.17 ± 1.75	6.00 ± 1.66	-2.06 ± 1.73	0.001
Intervention	8.09 ± 1.37	5.11 ± 1.60	-3.01 ± 1.9	0.0001
Independent t test	0.54	0.035	0.042	

The average age of pregnancy was 7.37 ± 0.83 weeks in women of the intervention group and 7.22 ± 0.8 weeks in women of control group.

Besides, the average number of pregnancy of women in the intervention and control groups was reported to be 2.80 ± 2.13 and 2.30 ± 1.43, respectively. Furthermore, 100% of women in the control group and 92.5% of women in the intervention group actually intended to be pregnant. No significant difference was observed between the intervention and control groups in terms of the individual variables mentioned above (p > 0.05).

In relation to the main purpose of this study, as shown in table 3, the findings prove that the average value of HG of women in the intervention group decreased from 8.09 ± 1.37 (prior to receiving the psycho-education) to 5.11 ± 1.60 (post-intervention), and in control group it decreased from 8.17 ± 1.75 (prior to intervention) to 6.00 ± 1.66 (post-intervention). Mean changes in the value of HG was -3.01 ± 1.9 in women of the intervention group and -2.06 ± 1.73 in women of the control group. Independent t-test also revealed that the average value of nausea and vomiting of pregnant women in the intervention group and the control group makes a significant difference after receiving psycho-education (p = 0.035). Additionally, the mean changes in the value of nausea and vomiting in the pregnant women of the two groups demonstrate a significant difference (p = 0.024). Paired t-test also confirmed that the average value of nausea and vomiting of pregnant women in both groups declined significantly in the posttest as compared to the pretest (p = 0.001).

## Discussion

This study was conducted to determine the effectiveness of psycho-education on the severity of nausea and vomiting in pregnant women who referred to urban general health centers. The results illustrate that HG severity in the women of intervention group who received psycho-education was significantly lower than in women of the control group. The results prove that psycho-education based on relaxation methods can effectively reduce HG severity. Studies that examine non-pharmacological treatments to moderate HG severity rarely integrate educational and therapeutic approaches, and they tend to focus on other approaches such as acupuncture (Gurkanand Arsalan 2008) (17). In this regard, one may refer to the study by Isbirand Mate (2016) which was designed to examine the effects of therapy (one session of face-to-face therapy and other instructions presented by conversations over the phone) on HG. The results of their research are in accordance with the present study and verify that HG severity is by far lower in the group that received therapy as compared to the control group. However, this reduction was not significant in the case of women with more severe forms of HG (8). Inasmuch as only women with medium severity of HG were qualified to enter the present study, the results appear to reconfirm the efficiency of educational and therapeutic approaches on reducing HG severity. In this regard, Clark et al. (2014) believe that most non-pharmacological approaches are efficient in treating morning sickness, but more severe forms of HG require seeing a doctor, taking medications, or even hospitalization (5).

In another study by Isbirand Mate (2016), the bulk of intervention and therapeutic recommendations was based on Roy adaptation model (8); however, the present study focuses on psycho-education, training and employment of PMR methods and guided imagery.

To analyze the results of the present study and the efficacy of psycho-education based on PMR and guided imagery techniques, it can be stated that, on the one hand, based on the findings of many researchers including Kuo et al. (2007), Nikibakhsh et al. (2016), Zinbarg et al. (2008), and Köken et al. (2008) HG severity is directly and significantly related to the stress and anxiety level in pregnant women, so that even as stress and anxiety of pregnant women intensify, HG becomes more severe, and vice versa (9, 11, 12, 23). On the other hand, the results of numerous researchers such as Nasiri et al. (2018), Jallo et al. (2015), and Charalambous et al. (2016) demonstrate that PMR and guided imagery techniques help reduce pregnant women’s stress and anxiety (28, 29, 18). Jallo et al. (2015) reported that in order to reduce stress and its related symptoms, guided imagery technique make it possible for the individual to communicate with her physical body so that her welfare and tranquility improve, and her negative emotional reactions to stress decrease (29). HG could be considered a type of negative emotional response of pregnant women to their underlying anxieties (9). In accordance with this analysis, the results of Charalambous et al. (2016) corroborate that the combination of guided imagery and PMR techniques together can bring about a significant reduction in a number of stress-related symptoms, including nausea and vomiting, in cancer patients receiving chemotherapy (19). In general, applying PMR and guided imagery techniques in from of psycho-education in the present study might have alleviated stress and anxiety levels in pregnant women. This psychological change appears, in turn, to have led to the moderation of HG severity. In agreement with these results, Kuo et al. (2007) state that to reduce pregnancy nausea and vomiting, it is necessary to focus on reducing the pregnant women’s stress and anxiety while enhancing their adaptability (23). 

The intervention of the present study involved a psychological aspect as well, which took into account self-expression, recognition of emotions and thoughts, acceptance of emotions, and improvement of positive emotions that help to accept pregnancy. Isbirand Mate (2010) believe that the sympathy of nurses and midwives [towards patients] improves the results of care interventions. Therefore, using empathic communication skills and allowing emotional expression is highly advised in the pregnant women with HG (6). In addition to lifestyle education and stress-reduction techniques, the present study observed that applying cognitive therapy and psychotherapy seems to be particularly effective in moderating the severity of HG. In this regard, the clinical trial of Faramarziet al. (2015) indicates that the combination of psychotherapeutic interventions and medical treatments, as opposed to medical treatments alone, is significantly more effective in resolving pregnancy problems such as nausea and vomiting symptoms, anxiety, depression, and pregnancy stress one month after the intervention (16). It could be inferred that interventions which involve psychological dimensions, such as the one used in the present study, improve mental health status of pregnant women, which, in turn, leads to lowering HG severity. Dehkordi et al. (2013) reported that HG must be considered in relation to women’s psychological and social status, because women’s psychological condition aggravates the severity of nausea and vomiting and makes it more difficult to treat such symptoms (1). 

Part of the intervention in this study involves adjusting the underlying stimuli, identifying nausea and vomiting stimuli, and making small lifestyle changes. The moderation of nausea and vomiting severity could be attributed to such behavioral lifestyle changes and modifying eating habits as well. Thus, in their qualitative study based on an adaptation model developed according to the opinions of pregnant women with HG, Isbirand Mate (2013) indicated that coping with contextual stimuli, including mobility, odor, exhaustion, stress, and eating habits can help to reduce the severity of pregnant women’s nausea and vomiting (2).

Some of findings of the present study showed that HG severity of women in both groups (control and intervention) in the posttest was significantly lower than that in the pretest. Because it took more than 6 weeks to conduct this research (since the beginning of the research until the end of the intervention and the time of posttest), the observed fall in the severity of nausea and vomiting in women of the intervention group could be attributed to approaching the 16^th^ to 20^th^ week of pregnancy, a period in which pregnancy nausea generally decreases automatically (2, 3). However, the improvement of the intervention group was significantly greater than that in the control group due to the psycho-educational treatment. 

***Limitations:*** Designing and conducting a multi-dimensional intervention which emphasizes psychological factors and involves an individual, face-to-face implementation could be counted as the strength of the present research. Conversely, excluding highly severe forms of HG from the study population and not examining/comparing the time of total disappearance of HG in the two groups could be regarded as the limitations of this study.

## Conclusion

The intensive, multi-dimensional psycho-education designed in this research underscores progressive muscle relaxation methods and guided imagery, making small changes in lifestyle, and attending to pregnant women’s emotional needs. The results established its effectiveness in mitigating the severity of nausea and vomiting in pregnant women. Given that midwives and nurses usually provide pregnant women with some advice on how to cope with pregnancy nausea and vomiting - suggestions which involve herbal medicine and alternative treatment -, psycho- educational factors mentioned in this study can also be integrated into these instructions and other forms of pregnancy care in order to assist vulnerable women deal with symptoms of nausea and vomiting and enable them to go through their pregnancy with the least amount of stress and discomfort.
